# Supplementary material for: Small RNA interactome of pathogenic E. coli revealed through crosslinking of RNase E
Source: EMBO J. 2016 Nov 11;36(3):374–87. doi: 10.15252/embj.201694639 (PMC5286369; doi:10.15252/embj.201694639)
Supplement: Supplementary file 1 — Appendix [file EMBJ-36-374-s001.pdf]

APPENDIX

Table of contents

1. Supplementary Methods 2-7
    a. *In silico* analysis of RNase E sequencing data 2
    b. Scoring RNA-RNA interactions 4
    c. Functional annotation of the mRNA-target clusters for specific sRNAs using Gene Ontologies 5
    d. Confirmation of interactions using the sfGFP 2-plasmid system 6
    e. References 7

2. Figure S1 8

SUPPLEMENTARY METHODS

<u>*In silico* analysis of RNase E sequencing data.</u>

Analysis of RNase E-RNA binding sites (CRAC data). Analysis of RNase E binding site identified by UV-crosslinking was performed using the pyCRAC software package as previously described (4). Regions containing statistically significant clusters of reads were defined using pyClusterReads.py and filtering for >100 cDNAs with >5nt overlaps within the cluster. We then identified clusters with an FDR <= 0.05 using pyCalculateFDRs.py. Peak maxima were identified within these statistically significant regions with a peak height > 50 reads and peak width > 20 nucleotides (as per Helwak et al. 2013). We additionally filtered for peaks with height >100 reads and peak width >20 nucleotides; and for peaks (height>50, width>20) that are within 1kb of a Hfq binding site defined in our previous analysis (Tree *et al* 2014). These three files of peak locations are provided within the GEO record GSE77463.

Hfq binding sites were similarly extracted from our previous datasets (Tree *et al*, 2014) using pyCRAC in GTF format. Oligo(A) tails were identified as previously described (Wlotzka *et al*, 2011).

Cumulative plots of non-genomically encoded poly(A) tails, RNase E and Hfq binding sites. In our previous CRAC analysis of Hfq binding sites we were able to flatten Hfq read peaks into read clusters representing the total length of the Hfq binding peak. However, we found that RNase E binding sites were too long to flatten into read clusters (ie: reduce read depth to 1 or 0 to allow each peak to contribute equally) and retain meaningful information about the RNase E binding site when plotting cumulative binding around genomic features. To retain information about the read profile at binding sites in our cumulative analysis we extracted the read depth at RNase E binding sites +/- 1kb and expressed read depth as a fraction of the maximum (set to 1). We were then able to cumulatively plot RNase E binding around genomic features. We have labelled these plots "Normalised read clusters". Hfq binding sites were defined as sites where Hfq binding peaks were found in 3 replicate datasets (ref 2) and we examined 672 RNase E binding peaks that were within 1kb of a Hfq binding site. For these 672 RNase E binding peaks we extracted the Hfq, oligo(A), and RNase E read count data to create a binding profile centred around the RNase E binding peak (+/- 1kb). Hfq, oligo(A), and RNase E read depths were normalised to the maximum count (set to 1) within the binding profile and a cumulative score for each position with the +/- 1kb was determined by adding the scores of the 672 sites together. The maximum peak height was 169 for Hfq, 364 for RNase E, and 138 for oligo(A) tails. To plot cumulative Hfq, oligo(A), and RNase E binding profile on the same axis, the maximum for each binding profile was set to 1. Cumulative plots of normalised read depth are labelled 'normalised read clusters'.

Analysis of RNase E-RNA-RNA interaction data (CLASH analysis). CLASH RNA-RNA interaction data was extracted from sequencing datasets using the *hyb* package (Travis *et al*, 2014). The UCSC *E. coli* O157:H7 str. Sakai genome (http://microbes.ucsc.edu/cgi-bin/hgGateway?db=eschColi_O157H7)(GenBank NC_002695, NC_002128, NC_002127) was used as a reference sequence. The sequencing data was analysed using the follow settings:

hyb demultiplex M=1 code=BARCODES preprocess check detect db=SAKAI anti=1 analyse in=FASTQ

Where BARCODES describes the 5'linker sequences, SAKAI is the database generated by make_hyb_db, and FASTQ is the input fastq file.

The *hyb* analysis provides data on interaction strength of the read halves using the UNAfold suite, the position of the interaction, counts for the total number of unique reads (unique read sequence or random nucleotide sequence within the barcoded linker), and whether the hybrid was identified in the RNA1-RNA2 and RNA2-RNA1 orientations. These data were extracted from the *hyb* output using custom scripts to generate plots of attributes for the different RNA classes.

Annotation of hybrids. The *hyb* package provides coordinates for hybrid halves mapping to the *E. coli* O157:H7. Hybrid halves were assigned to a genomic feature using the *Escherichia coli* O157:H7 GTF annotation from UCSC Table browser (http://microbes.ucsc.edu) using custom scripts. We additionally added the location of previously predicted novel small RNAs in EHEC (EcOnc; ref 2). Untranslated regions were added to the annotation by extending CDS features 100nt 5' and 3' unless the UTR overlapped known features, where the UTR was extended to the feature. 5'UTRs were extended before 3'UTRs and do not overlap. Hybrids halves were first annotated with features in the UCSC GTF file, those that did not overlap a feature were annotated with UTRs, and the remaining hybrid halves were annotated with antisense features (annotated with 'as'feature name).

Identification of known interactions. To convert sRNATarBase 3.0 (Wang *et al.* 2015) *E. coli* K12 mRNA seed coordinates into *E. coli* O157:H7 str. Sakai coordinates, the seed sequences were padded on both sides with 100bp of their surrounding sequence from the K12 genome, using a custom Python script. If the location of the seed was missing from the database, the seed position was determined computationally. These calculated locations were then checked against the literature (Table EV4). In the case of other missing data or discrepancies between the database and the genome (e.g. seed sequence not found at listed location) the literature was also checked (Table EV4). BLAST v2.2.28+ was used to locate the padded seeds in the Sakai genome. Custom Awk and Python scripts were then used to tabulate the seed locations

in the Sakai genome from the BLAST results (Table EV3). Another custom Python script was used to convert the local (i.e. relative to TSS) coordinates from sRNATarBase to K12 genomic coordinates (Table EV3). The EHEC sRNA-mRNA seed interactions were verified using IntaRNA v1.2.5 (Busch *et al.* 2008).

To calculate the significance of recovering 14/125 experimentally verified sRNA-mRNA interactions, we used a Monte Carlo approach to determine the probability of randomly recovering a verified mRNA seed sequence with ANY sRNA. This provides an overestimate of probability of recovering an mRNA seed ligated to its cognate sRNA. We used shuffleBed (Bedtools) to randomize mRNA target positions and restricted the randomised coordinates to within RNase E binding sites. We then use intersectBed to determine whether the randomised mRNA targets overlapped an experimentally verified mRNA interaction site. For 7520 iterations (number of sRNA interactions recovered with any RNA) the maximum overlap with verified mRNA target sites was 9 (4 iterations). We calculated the probability of recovering 9 verified mRNA interaction sites using the formula $p=(r+1)/(n+1)$, where r is the number iterations that were >= the test value (4 iterations), and n is the total number of iterations (7520). The probability of randomly recovering 9 experimentally verified mRNA target sites with ANY sRNA is 6.6 $\times 10^{-4}$ indicating that the probability of recovering 14 verified mRNA target sites with the COGNATE sRNA is << 6.6 $\times 10^{-4}$.

Scoring RNA-RNA interactions.

In order to rank the reliability of sRNA-mRNA interactions, we scored interactions based on (1) the number of unique sequence reads corresponding to the interaction; 2) detection of the interaction in replicate datasets; 3) recovery of the hybrid sequences in both RNA1-RNA2 and RNA2-RNA1 orientations, indicating ligation at opposite ends of the duplex; 4) inclusion of a non-genomic encoded oligo(A) tail at the 3’ end of the target RNA sequence, which is indicative of sRNA directed cleavage and subsequent tailing; 5) overlap of both hybrid regions with Hfq binding sites determined by UV-crosslinking and indicating Hfq-dependence. The later criterion assumes that Hfq binds sufficiently close to the seed sequence to be included in Hfq-bound sequencing reads. Our earlier analysis of Hfq binding sites demonstrated that Hfq often intimately associates with the seed sequence (Tree *et al*. 2014) and with RNase E binding sites (Figure 1E). Further, Hfq is known to nucleate complementary seed regions at its lateral surface through electrostatic interactions with conserved arginine residues at positions R16, R17, and R19 (Panja *et al*. 2013). To verify that Hfq-dependent interactions were enriched by filtering our sRNA-mRNA interaction data we looked for retention of the 14 experimentally verified, Hfq-dependent sRNA-mRNA interactions identified in the RNase E-CLASH dataset. Known Hfq-dependent sRNA-mRNA pairs were enriched by filtering for interactions that overlap a Hfq binding site (12/14 verified sRNA-mRNA interactions, 850/1721 non-verified interactions, p=0.0068) indicating that this criterion provides useful functional information. We then looked at the cumulative distribution function of scores for

experimentally verified and non-verified interactions when ranking on the number of hybrids alone (Figure EV4B), the scoring criteria without Hfq binding data (without criteria 5, Figure EV4C), and using all five criteria (Figure EV4D). The addition of each scoring criteria improved the distribution of scores for the 14 verified sRNA-mRNA interactions when compared with non-verified interactions (total). We conclude that our scoring criteria provide significant separation of functional sRNA-mRNA interactions.

Probabilistic analysis of RNA-RNA interactions. To estimate the false discovery rate for RNA-RNA interactions, we compared each observed interaction with the background probability that the same ligation event could occur spuriously. The analysis was based on the method described in Sharma *et al.* (2016) [1]. Briefly, the probability of drawing a hybrid-half at random, $P(g_x)$, was estimated by the maximum read depth at the hybrid-half (non-hybrid reads) divided by the total number of mapped reads in the dataset (N). The background probability for observing an RNA-RNA interaction, $pdf(g_x, g_y)$, was estimated by multiplying the probabilities $P(g_x)$ and $P(g_y)$. Ligation events that were not observed were set to a probability of zero, as per Sharma *et al.* (2016). The probability distribution $pdf(g_x, g_y)$ was re-normalized to the sum of 1. The background probability of observing k occurrences of interaction between g_x and g_y was modeled using the binomial distribution, $k \sim Binomial(p=pdf(g_x, g_y), N)$. These calculations were used to assign a p-value to each experimentally observed pair of RNA-RNA interaction. The p-values were adjusted using Benjamini-Hochberg multiple testing corrections, before applying a false discovery rate threshold of p-value < 0.05. For RNA-RNA interactions that were observed in both of the two replicates, the corrected p-value was combined using the Fisher's method.

Motif analysis within sRNA-mRNA interactions. For each sRNA with hybrid sRNA-target RNA sequences identified in the *hyb* output file, the corresponding RNA interaction sequence was extracted from the genomic sequence. For sRNAs with >=10 interactions, MEME (Bailey & Elkan, 1994) was used to identify an enriched motif. The sequence logo for the motif was then used to identify complementary sequences within the sRNA using FIMO (Grant *et al*, 2011). Complementary sRNA sequences and target RNA motifs with an e-value < 10^{-4} are presented in Appendix Figure S1.

<u>*Functional annotation of the mRNA-target clusters for specific sRNAs using Gene Ontologies*</u>

Gene ontology (GO) is a controlled vocabulary used to unify the representation of gene and gene product attributes across all species. We have used GO annotations and enrichment of GO categories for mRNA target clusters belonging to specific sRNAs, to get a indication of the possible function of the sRNA. NCBI repositories contain thousands of sequenced *E. coli* strains but gene ontology annotations were found to be available for a selected few, not including *E. coli* O157:H7 str. Sakai. Hence the first step was to get a detailed gene ontology

annotation for *E. coli* O157. The reference strain K12 for which detailed GO annotations were found available, and mapping the ontologies from this strain would result in loss of data for the O157 specific genes. Hence, the *E. coli* O157 gene set with 5361 genes were put through the Blast2GO pipeline (8): a bioinformatics platform for high-quality functional annotation and analysis of genomic datasets. Gene ontologies were retrieved for 4135 genes across the three ontology categories: Biological process, molecular function and cellular localization. The mRNA target clusters for individual sRNAs were then analyzed for GO enrichments using the tool BiNGO (9). BINGO is a Cytoscape plugin to assess overrepresentation of Gene Ontology categories in Biological Networks. BiNGO mapped the predominant functional themes of the tested gene sets on the GO hierarchy. An additional step, which involved removal of redundancies in the GO structure, was applied using the tool REVIGO (10). REVIGO is a web server which summarizes long, unintelligible lists of GO terms by finding a representative subset of the terms using a simple clustering algorithm that relies on semantic similarity measures. Customized python scripts were also developed at various steps in the analysis process for generating input datasets that were compatible with the tools.

Confirmation of interactions using the sfGFP 2-plasmid system.

Flow cytometry to count sfGFP-expression . Flow cytometry to count sfGFP-expression. The translation of sGFP was monitored in the presence of constitutively transcribed sRNAs using a FACSCanto II flow cytometry system (BD). Acquisition of sample data was performed using the built-in Diva (FACSCanto II) software. Fluorescence of GFP was quantified using a 530/30 nm bandpass filter. FSC and SSC of cells were used to gate the bacterial population. A minimum of 100, 000 gated events were collected for each sample. Data analyses were performed with FlowJo software to calculate Median Flouresence Intensity (MFI).

For flow cytometric analysis, DH5α strains were grown overnight in filtered LB culture. Samples were additionally analyzed in a Top10F' background. Strains were grown to 0.6 OD_{600} and maximally induced with IPTG (1mM) and anhydrotetracycline hydrochloride (200nM, Sigma) for one hour. Cells were diluted 1:10 in 0.22 μm filtered PBS. All sorting analyses were carried out in triplicate. Control cultures expressing GFP and Lux were used as positive and negative controls respectively.

RT-QPCR analysis of superfolder GFP transcript abundance. Total RNA isolation was performed for three biological replica per mRNA-GFP:sRNA pair using the RNeasy® Mini Kit (Qiagen), in accordance with the manufacturer's instructions, including on-column DNAseI treatment. RNA purity and concentration were determined using a NanoDrop 2000 Spectrophotometer (Thermo Scientific). 1ug of RNA from each sample was reverse transcribed using the Superscript III first-strand synthesis system (Thermofisher Scientific) as recommended by the supplier. Quantitative PCR technical triplicate reactions were prepared in 96-well optical plates with 2 μL diluted cDNA sample, using KAPA SYBR Fast Universal

qPCR kit according to the manufacturer's recommendations. sfGFP expression was amplified with Forward primer- 5′GTTCCATGGCCAACACTTGTCACT 3′ and Reverse primer, 5′TACATAACCTTCGGGCATGGCACT3′) as described in Hersch *et al* 2013. 16S rRNA gene (Forward primer, 5′-ATTGACGTTACCCGCAGAAG-3′, Reverse primer, 5′ CGCTTTACGCCCAGTAATTC 3′) expression was used as control for normalization. Amplification was carried out in an ABI thermocycler (Australia). Melting curves were generated for each gene to ensure the purity of the amplification product. sf*GFP* expression was determined by relative quantification, which was calculated using 2 e(−ΔΔCt) method.

SUPPLEMENTARY METHODS REFERENCES

1. Helwak A, Kudla G, Dudnakova T, Tollervey D (2013) Mapping the human miRNA interactome by CLASH reveals frequent noncanonical binding. *Cell* 153(3):654–665.
2. Travis AJ, Moody J, Helwak A, Tollervey D, Kudla G (2014) Hyb: A bioinformatics pipeline for the analysis of CLASH (crosslinking, ligation and sequencing of hybrids) data. *Methods* 65(3):263–273.
3. Wlotzka W, Kudla G, Granneman S, Tollervey D (2011) The nuclear RNA polymerase II surveillance system targets polymerase III transcripts. *EMBO J* 30(9):1790–1803.
4. Tree JJ, Granneman S, McAteer SP, Tollervey D, Gally DL (2014) Identification of Bacteriophage-Encoded Anti-sRNAs in Pathogenic Escherichia coli. *Mol Cell* 55(2):199–213.
5. Bailey TL, Elkan C (1994) Fitting a mixture model by expectation maximization to discover motifs in biopolymers. *Proc Int Conf Intell Syst Mol Biol* 2:28–36.
6. Grant CE, Bailey TL, Noble WS (2011) FIMO: scanning for occurrences of a given motif. *Bioinformatics* 27(7):1017–8.
7. Brickman TJ, Armstrong SK (1996) Colicins B and Ia as novel counterselective agents in interspecies conjugal DNA transfers from colicin-sensitive escherichia coli donors to other gram-negative recipient species. *Gene* 178(1- 2):39–42.
8. Conesa A, Gotz S, Garcia-Gomez JM, Terol J, Talon M, et al. (2005) Blast2GO: a universal tool for annotation, visualization and analysis in functional genomics research. Bioinformatics 21: 3674-3676.
9. Maere S, Heymans K, Kuiper M (2005) BiNGO: a Cytoscape plugin to assess overrepresentation of gene ontology categories in biological networks. Bioinformatics 21: 3448-3449.
10. Supek F, Bosnjak M, Skunca N, Smuc T (2011) REVIGO summarizes and visualizes long lists of gene ontology terms. PLoS One 6: e21800.

Appendix Figure S1

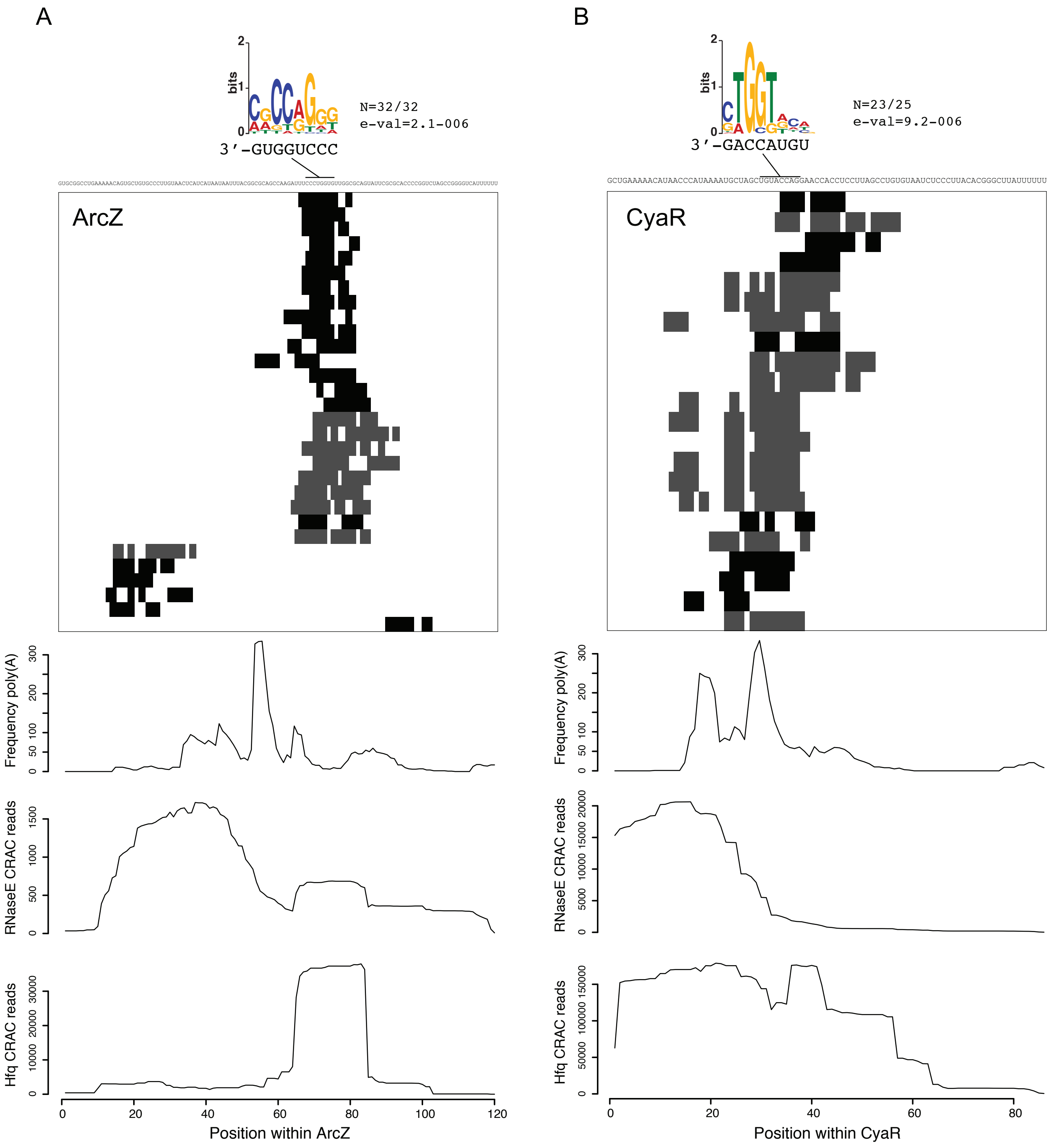

Appendix Figure S1 continued

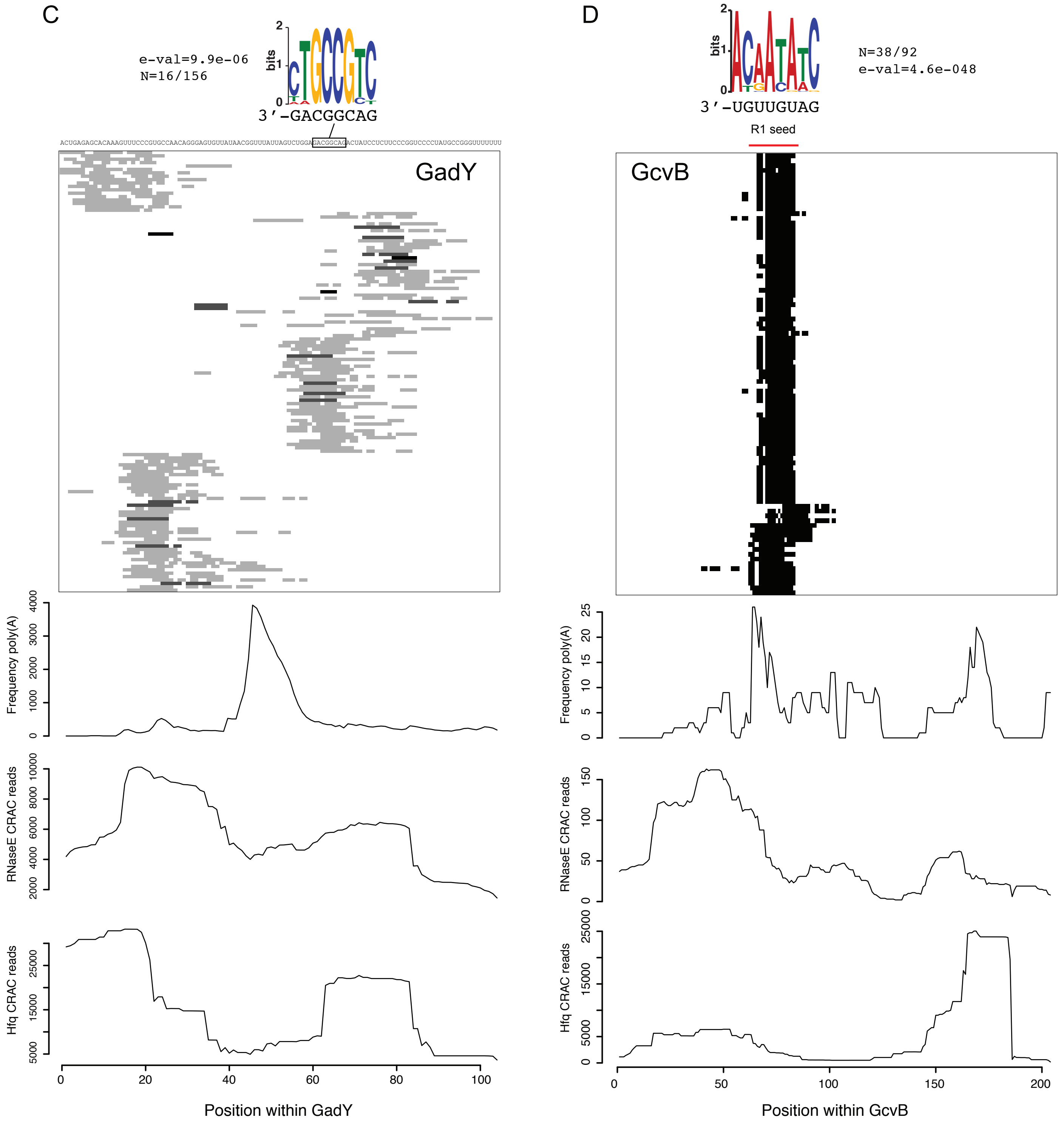

Appendix Figure S1 continued

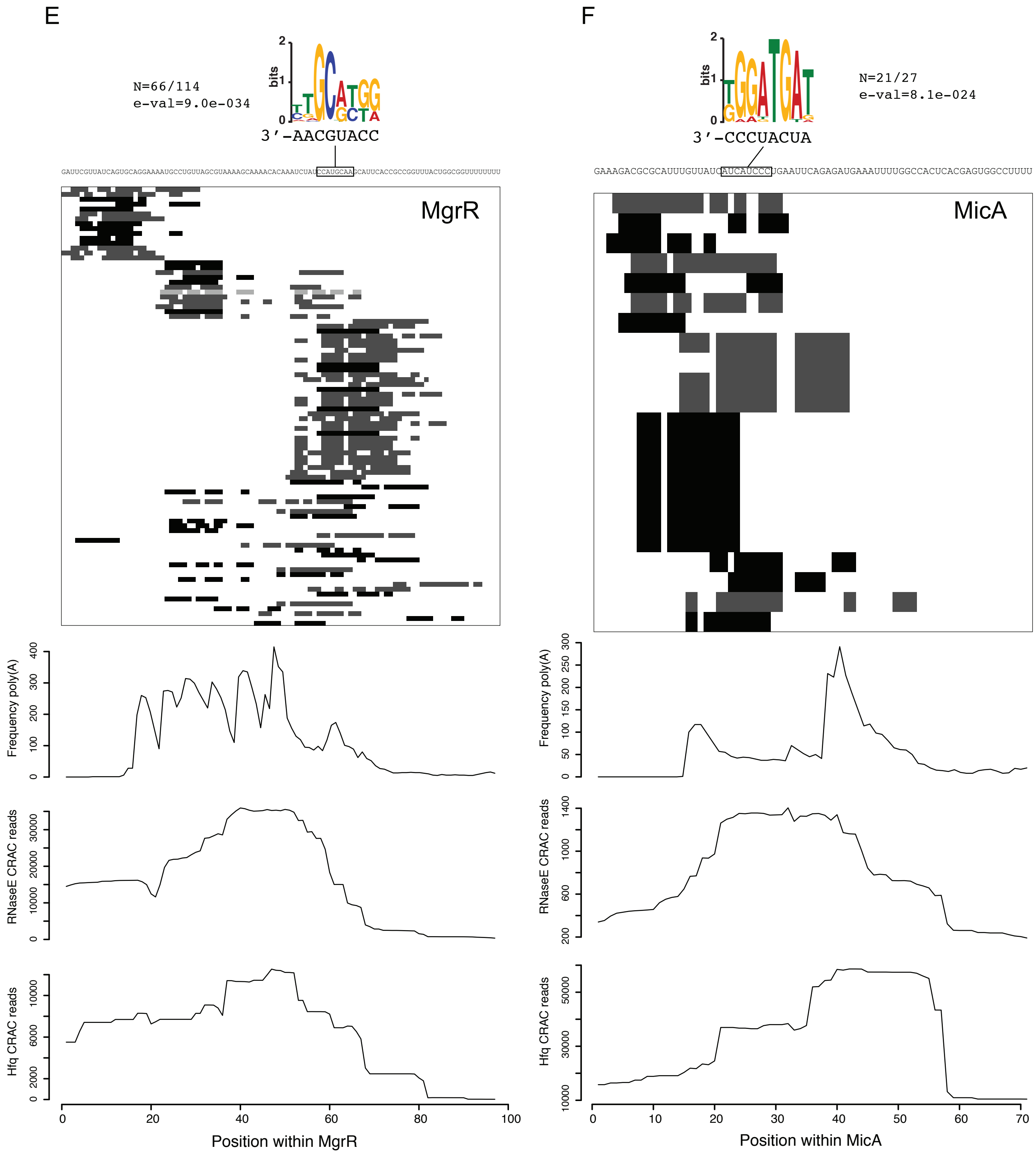

Appendix Figure S1 continued

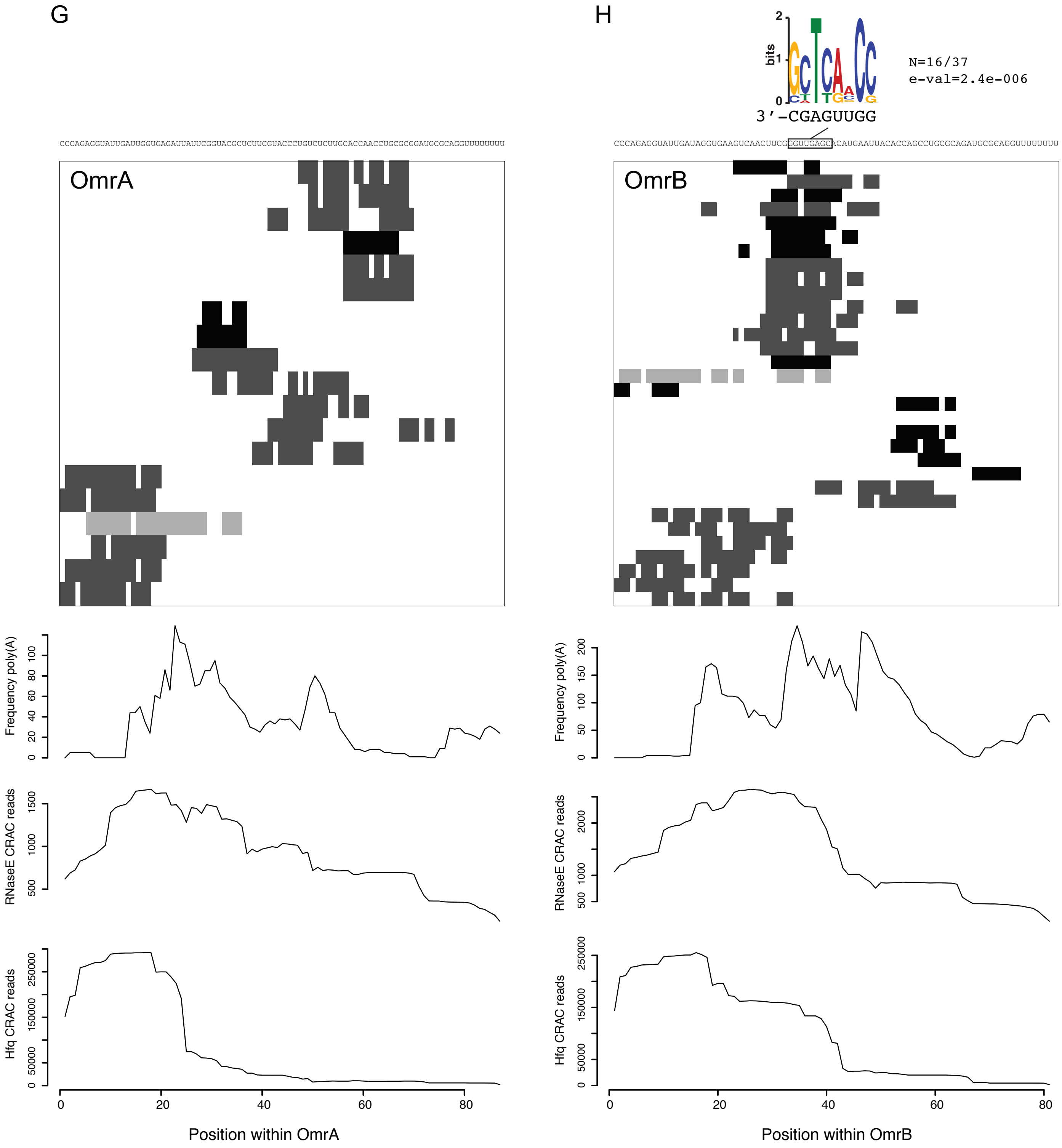

Appendix Figure S1 continued

I

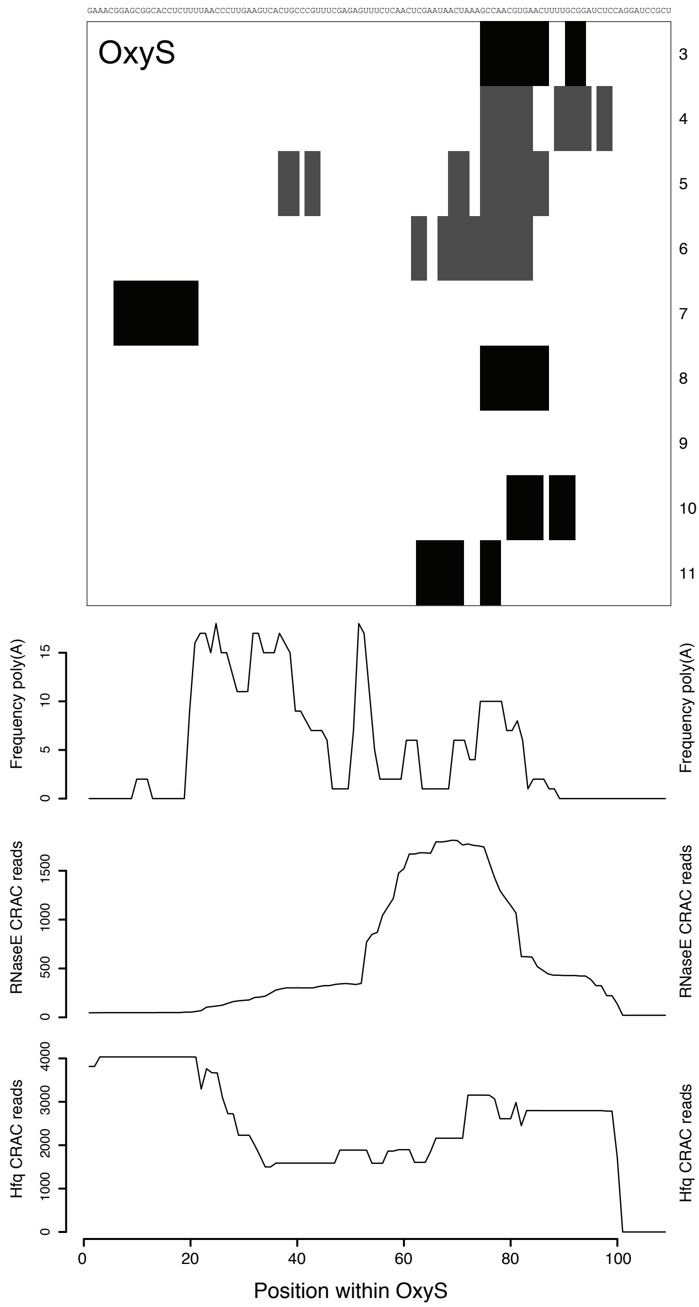

J

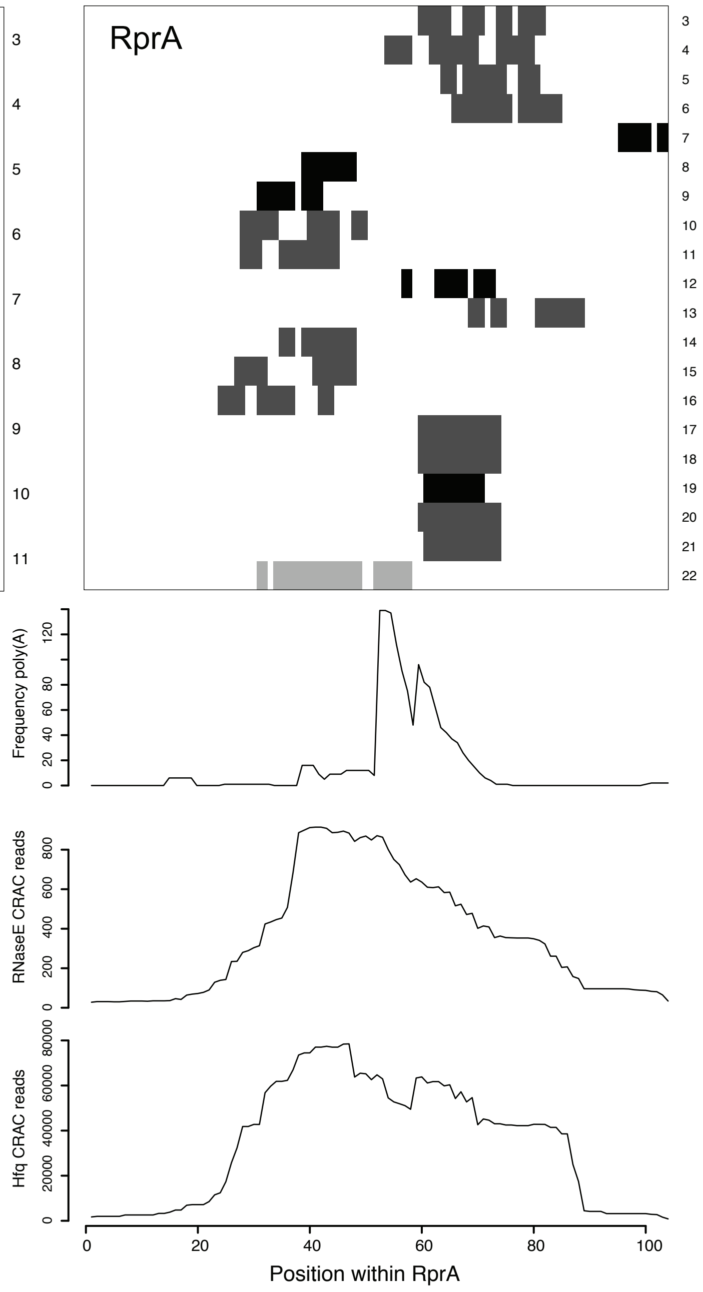

Appendix Figure S1 continued

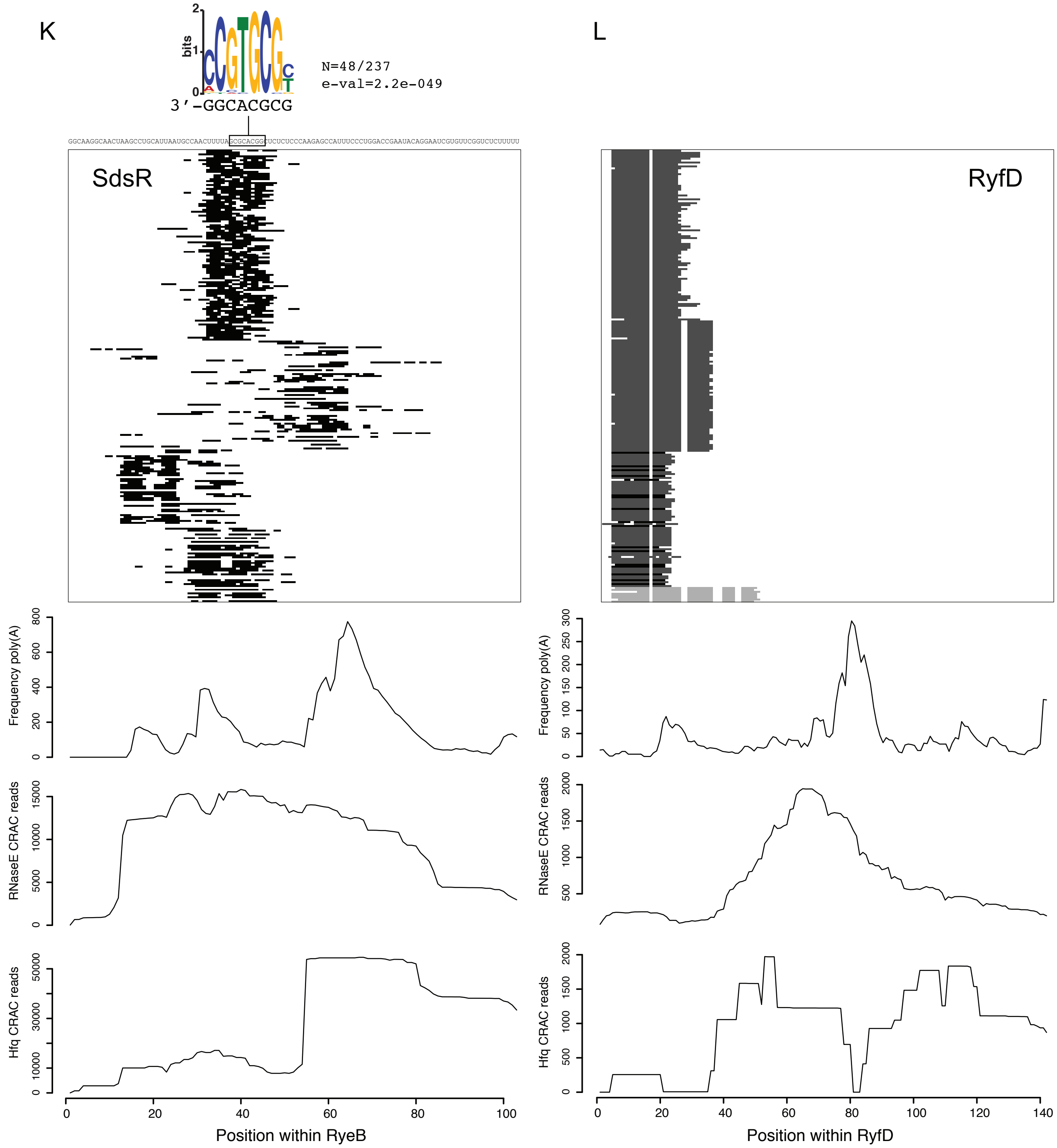

Appendix Figure S1 continued

M

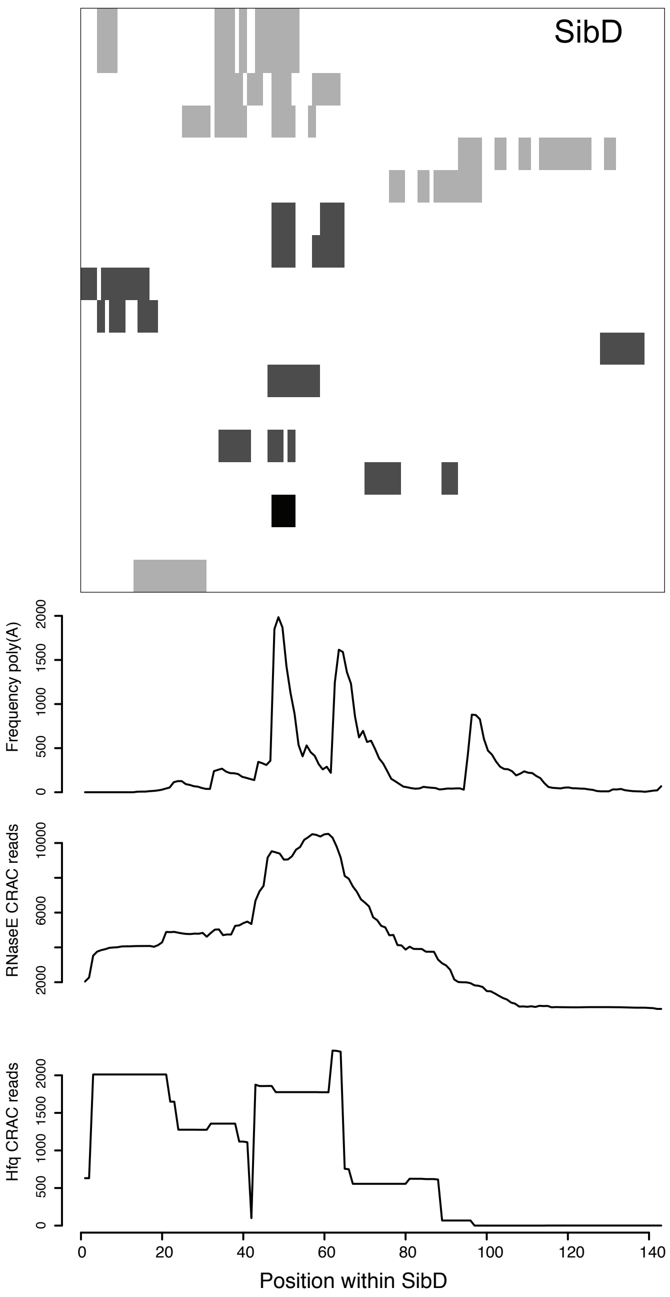

N

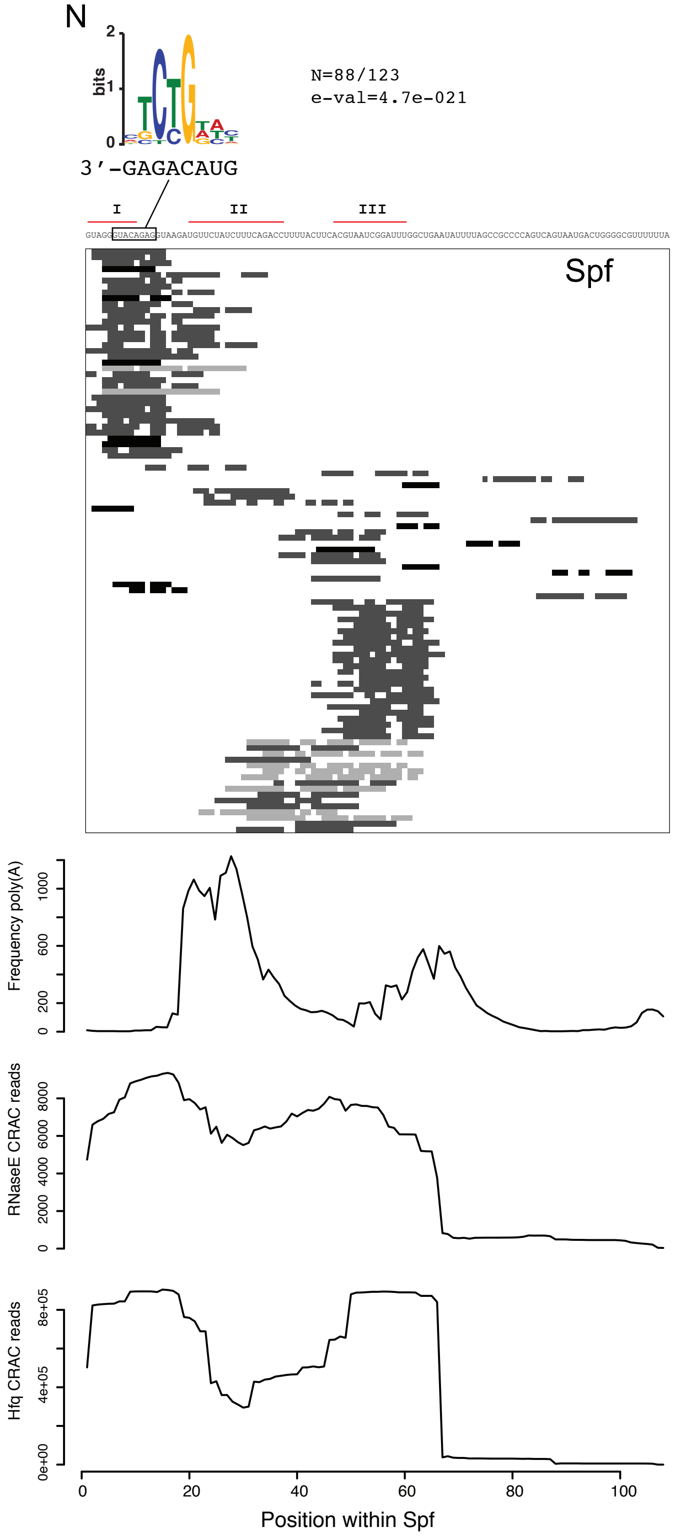

Appendix Figure S1 continued

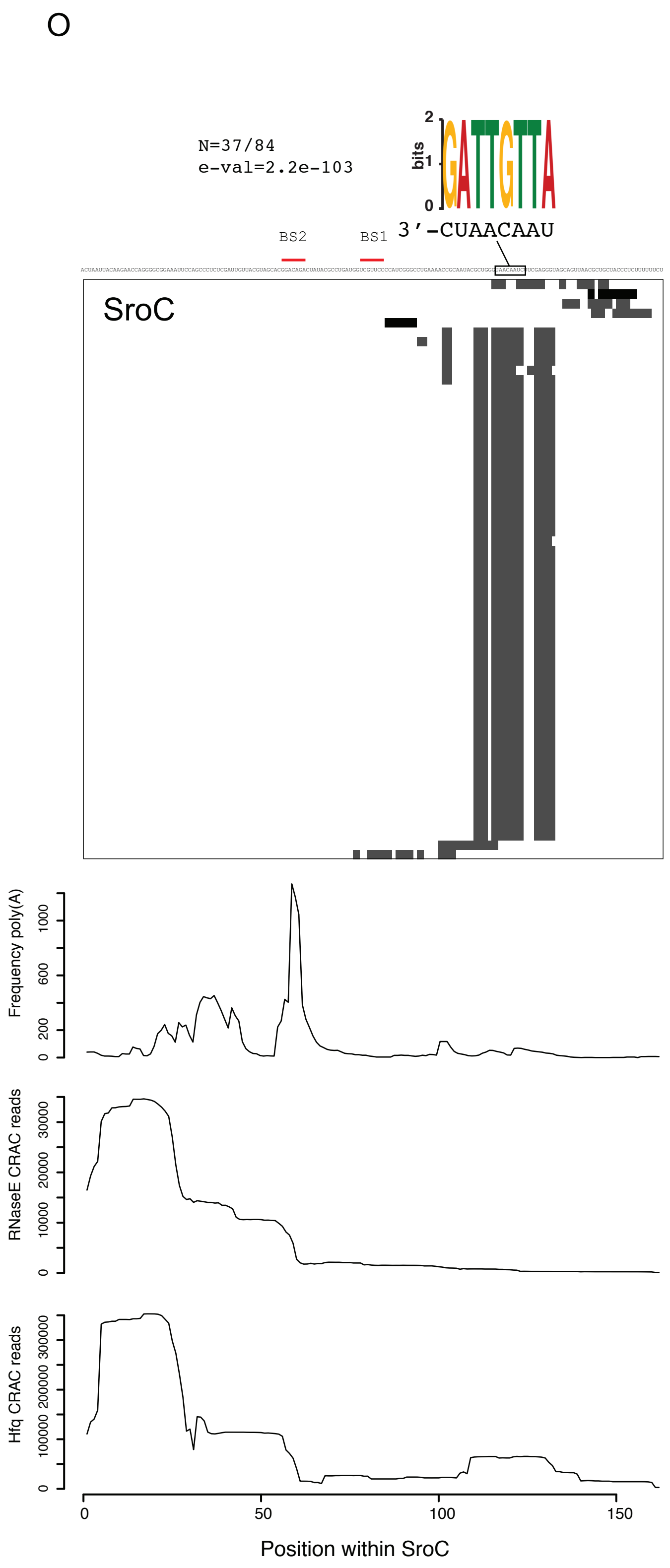

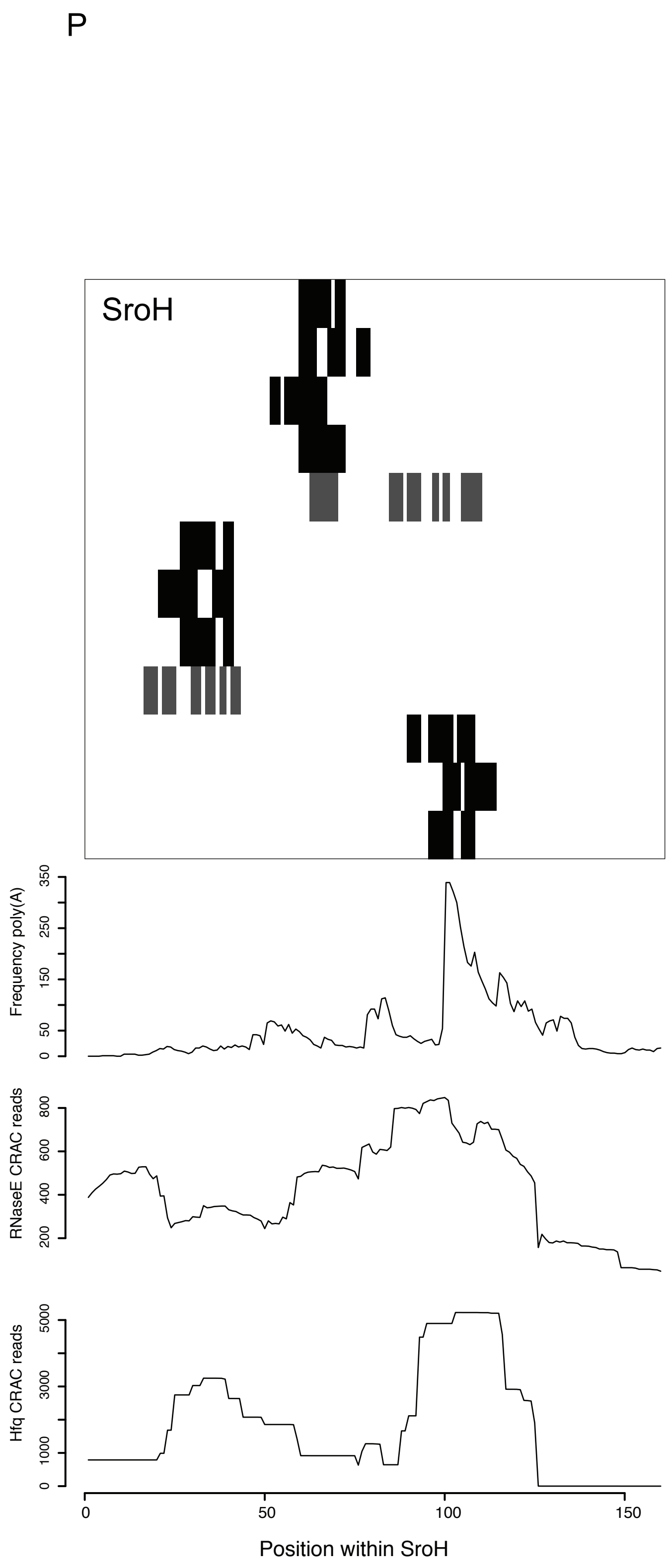

Appendix Figure S1. Target RNA interactions define small RNA seed regions. For each sRNA (indicated within the heatmap) the sequence is given (top) and the position of predicted basepairing with target RNAs identified by RNase E-CLASH is shown (heatmap of baseparing strength along sRNA for each mRNA target). Binding sites for Hfq, RNase E, and non-genomic encoded oligo(A) tails (determined by CRAC) are plotted for the corresponding regions as line plots below the heatmap. Motifs that were enriched in the target RNA were identified using MEME and are shown above the heatmap with the proportion of target RNAs carrying the motif (N) and the expected value for the motif (e-val). Complementary sequences within the sRNA were identified using FIMO and are shown below the motif. Positions of complementary sequences are boxed.
