# Supplementary material for: Small RNA interactome of pathogenic E. coli revealed through crosslinking of RNase E
Source: EMBO J. 2016 Nov 11;36(3):374–87. doi: 10.15252/embj.201694639 (PMC5286369; doi:10.15252/embj.201694639)
Supplement: Supplementary file 2 — Expanded View Figures PDF [file EMBJ-36-374-s002.pdf]

Expanded View Figures

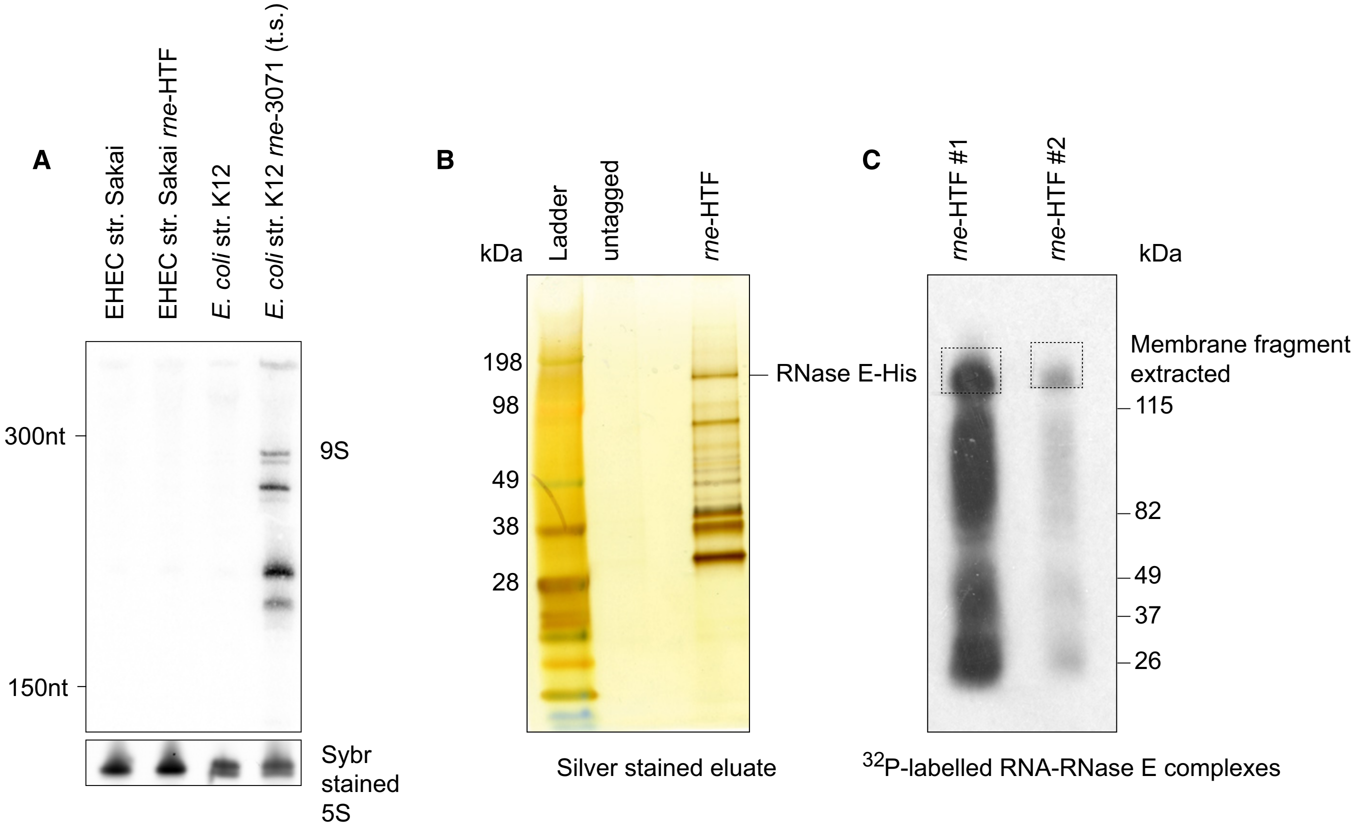

Figure EV1. C-terminally tagged RNase E is functional and facilitates purification of labelled RNA–RNase E complexes.

A RNase E is required for processing of 9S rRNA into mature 5S rRNA, and we assessed 5S rRNA processing in our His-FLAG-tagged RNase E strain. Enterohaemorrhagic *E. coli* (EHEC) O157:H7 str. Sakai and the isogenic *rne-HTF* insertion mutant were grown to an OD_{600} of 0.6 in LB broth at 37°C and total RNA was harvested. To compare wild-type and impaired 5S rRNA processing, we additionally cultured *E. coli* str. K12 (N3433) and the isogenic temperature-sensitive *rne-3071* mutant (N3431) to OD_{600} 0.6. The *rne-3071* mutant was temperature-shifted to 43°C for 30 min and total RNA harvested. 1 μg of total RNA was separated on an 8% TBE–urea polyacrylamide gel and blotted for 9S rRNA. Mature 5S rRNA is shown in the SYBR-stained loading control below.

B RNA–protein complexes were eluted from Ni-NTA resin (see Materials and Methods) and precipitated and 50% of the eluate separated on a NuPAGE Bis-Tris 4–12% gel and silver-stained. *E. coli* O157:H7 str. Sakai (untagged; negative control) and the isogenic *rne-HTF* mutant were analysed for stringency of the dual-affinity purification.

C Replicate [^{32}P]-labelled RNA–RNase E complexes were separated on NuPAGE Bis-Tris 4–12% gradient gels and transferred to nitrocellulose membranes. The labelled complexes were imaged by autoradiography and complexes with a molecular mass equivalent to RNase E–RNA fragments (dashed boxes) were excised from the membrane (see Materials and Methods).

Source data are available online for this figure.

© 2016 The Authors

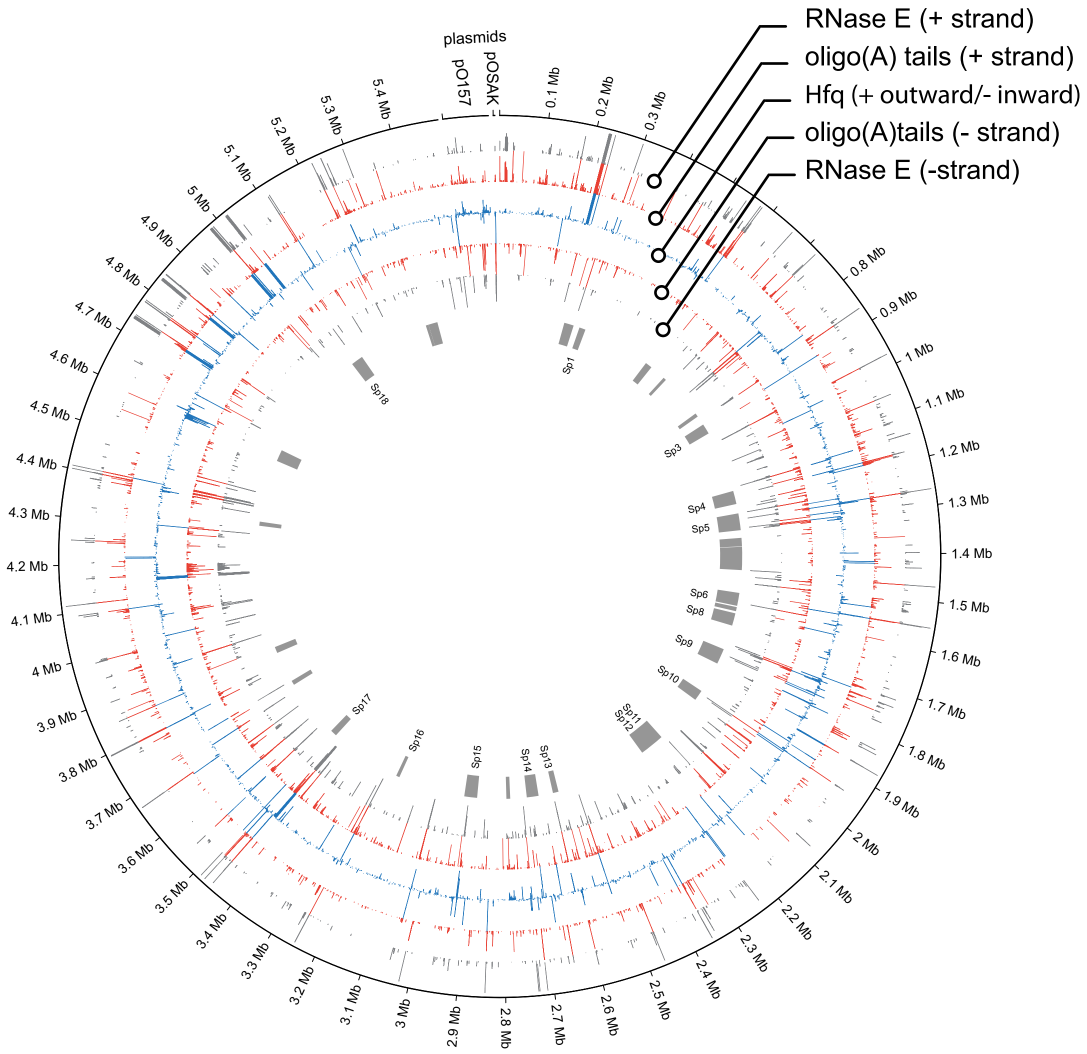

Figure EV2. Circos plot of RNase E binding sites (grey), non-genomically encoded oligoadenylation sites (red), and Hfq binding sites (blue).
Positive strand binding sites are shown in the outer rings and negative strand binding in the inner rings. Pathogenicity islands (S-loops [Sp]) are shown in grey (centre).

 © 2016 The Authors

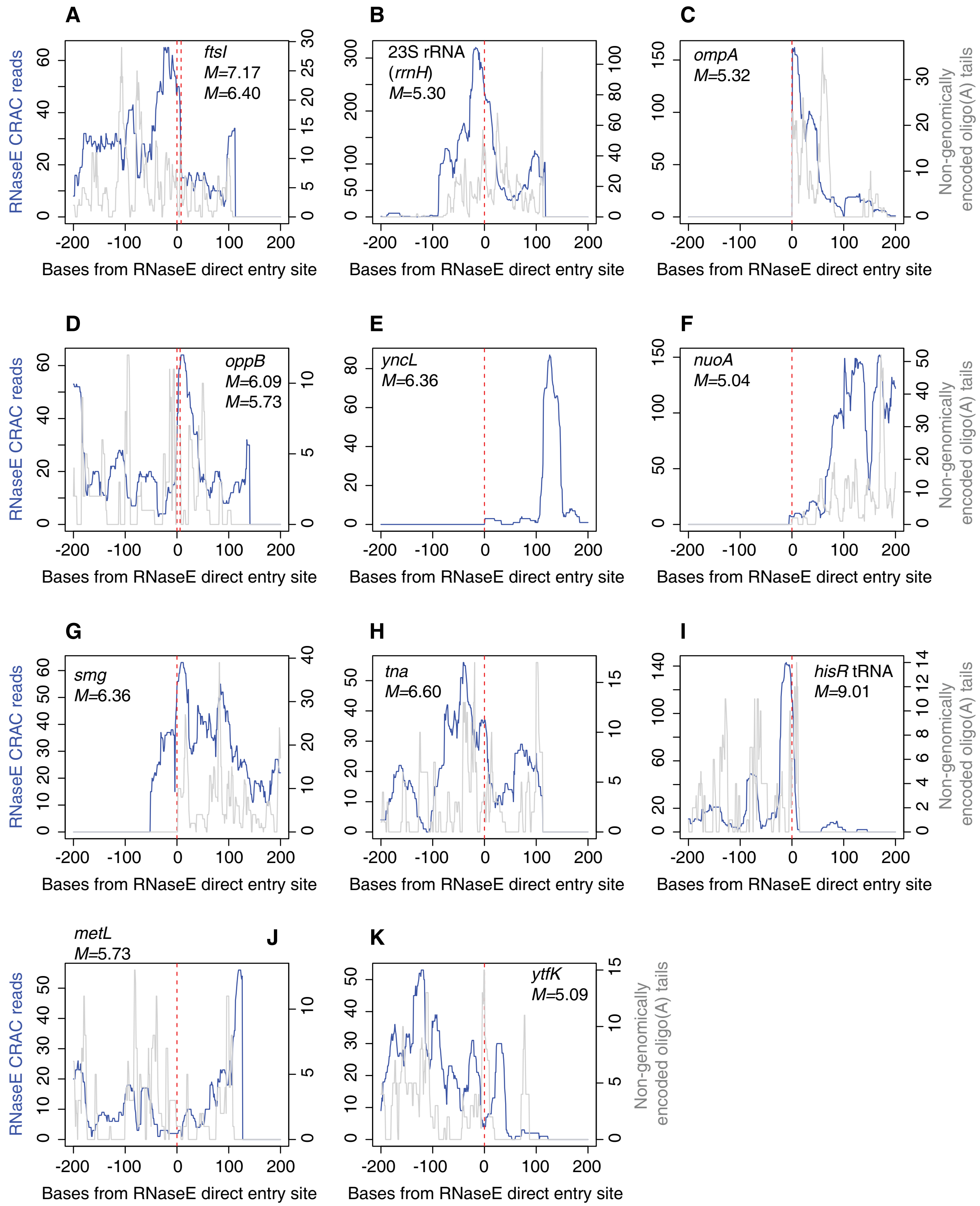

Figure EV3. RNase E binding and oligoadenylation at RNase E direct entry sites.

A–K Direct entry sites identified by Clarke *et al* (2014) were screened for peaks > 50 reads ± 200 nt from the direct entry site in our EHEC RNase E crosslinking dataset #1. RNase E binding (blue) and oligoadenylation (grey) are shown. Gene names, enrichment scores (*M*) and coordinates for direct entry sites were taken from Clarke *et al* (2014). For panels (A and D), two closely associated direct entry sites are indicated on the same plot.

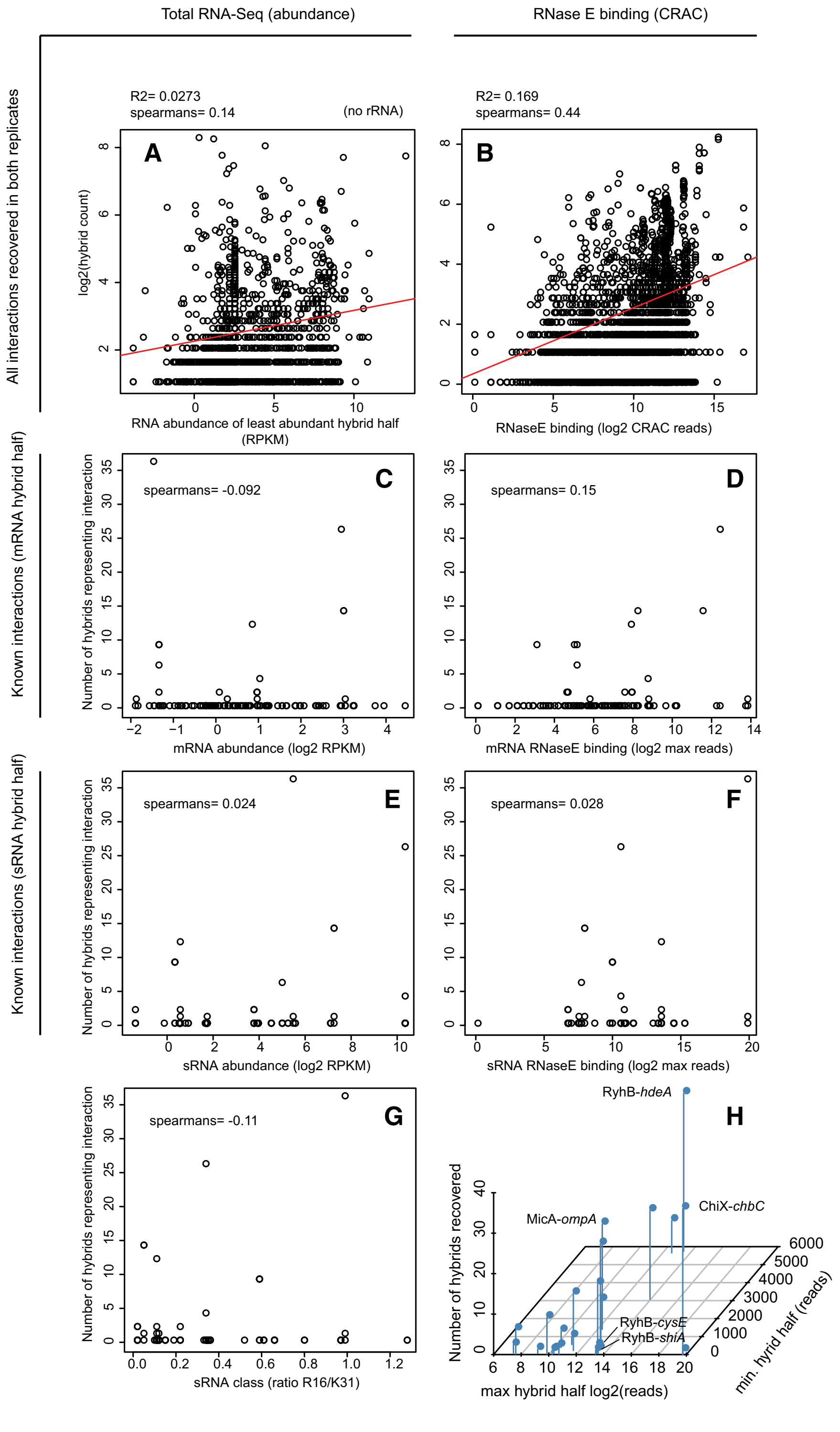

Figure EV4. Correlations between recovery of hybrids, total RNA abundance and RNase E crosslinking.

For each hybrid in the dataset, the RNA abundance (assessed by total RNA-Seq) and RNase E binding (RNase E CRAC) at each hybrid half were determined.

- A, B For all interactions recovered in both replicates, the number of hybrid reads mapping to an interaction was correlated with RNA abundance (of the least abundant hybrid half; A), and RNase E crosslinking (also for the lowest hybrid half; B).
- C–F The number of hybrids recovered for 125 experimentally verified sRNA–mRNA interactions was also correlated with RNA abundance at the target (C) and sRNA (E), and RNase E crosslinking at the mRNA (D) and sRNA (F).
- G For each known sRNA–mRNA interaction, the number of hybrids recovered was also plotted against the ratio of HfqR16/HfqK31 IP (previously determined by Schu *et al* 2015). The HfqR16/HfqK31 ratio is indicative of sRNA class where Class I $<$ 1 $<$ Class II sRNAs. For each plot, the Spearman correlation between the variables is shown.
- H RNase E crosslinking and hybrid recovery for known sRNA–mRNA interactions including those described in Fig 5. The number of hybrids representing an interaction (*z*-axis) was plotted against RNase E crosslinking for the lower (*y*-axis) and higher (*x*-axis) crosslinked halves.

 © 2016 The Authors

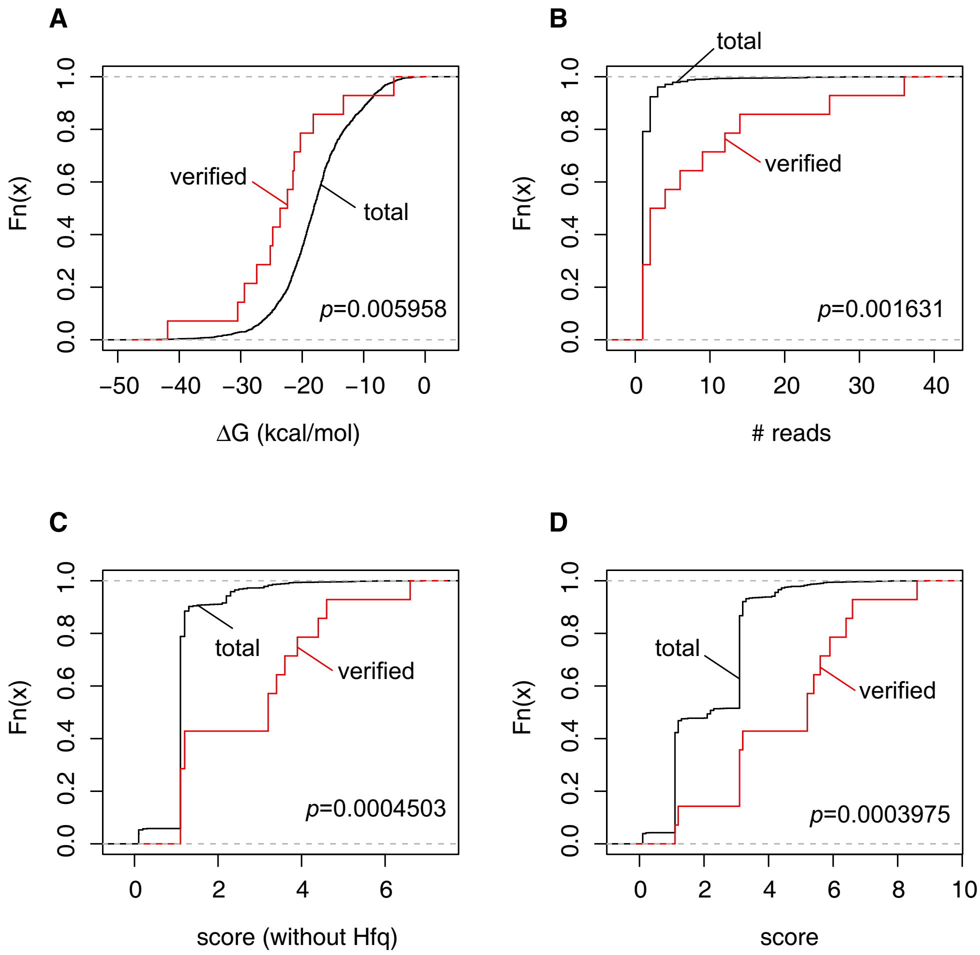

Figure EV5. To determine whether the scoring criteria provided a useful ranking of functional sRNA–mRNA interactions, we looked at the distribution of scores assigned to 14 experimentally verified sRNA–mRNA interactions (see Table EV5).

A–D Cumulative distribution functions for the total (unverified sRNA–mRNA) interactions (black) and for verified sRNA–mRNA interactions. Verified sRNA–mRNA interactions were found to have a significantly lower distribution of interaction strength (free energy of interaction, ΔG; panel A). For our scoring criteria, we looked the effectiveness of ranking interactions by the number of unique reads recovered (B), by applying scoring criteria 1–4 (i.e. excluding an overlap with a Hfq binding site) (C), or for scoring interactions on all five criteria (D). We applied a two-sample Kolmogorov–Smirnov test to each CDF (*p* indicated in plot) and found that scoring interactions on all five criteria gave the most significant separation of verified sRNA–mRNA interactions.

© 2016 The Authors
