# Supplementary material for: Small RNA interactome of pathogenic E. coli revealed through crosslinking of RNase E
Source: EMBO J. 2016 Nov 11;36(3):374–87. doi: 10.15252/embj.201694639 (PMC5286369; doi:10.15252/embj.201694639)
Supplement: Supplementary file 3 — Table EV1 [file EMBJ-36-374-s004.docx]

Expanded View Table 1: sRNA-mRNA interactions are recovered in Hfq- and RNaseE-CLASH data

| Protein | Dataset | # total reads | # total mapped (non-hybrid) reads | # hybrids interactions | # sRNA-mRNA interactions* | # known sRNA-mRNA interactions** | Percent recovery interactions/reads (sRNA-mRNA interactions /total interactions) |
| --- | --- | --- | --- | --- | --- | --- | --- |
| Hfq† | 1 | 36,174,905 | 32,320,299 | 1399 | 53 | 0 | 0.004% (3.7%) |
|  | 2 | 32,766,249 | 26,608,069 | 472 | 10 | 0 | 0.0018% (2.1%) |
|  | 3 | 12,626,377 | 10,204,035 | 109 | 2 | 0 | 0.0011% (1.8%) |
|  | 4 | 4,200,656 | 3,602,372 | 54 | 2 | 0 | 0.0015% (3.7%) |
|  | 5 | 63,943,583 | 58,847,737 | 3870 | 221 | 2 | 0.0066% (5.7%) |
|  |  |  |  |  |  |  |  |
| RNase E | 1 | 18,224,008 | 11,649,382 | 168,678 | 1750‡ | 14 | 1.44% (1.03%) |
|  | 2 | 3,678,239 | 2,152,636 | 17,851 | 194‡ | 3 | 0.83% (1.08%) |

*mRNA includes 50nt of 5’ and 3’ UTR.

**From 125 known interactions defined by sRNATarBase3.0

†Datasets described in Tree *et al* Mol Cell 2014

‡ Fewer hybrids are found in the cumulative dataset presented in Expanded View Table 3 as a single hybrid may bridge more than on hybrid in the replicate dataset. We present 1733 in EV Table 3.
