# Supplementary material for: Small RNA interactome of pathogenic E. coli revealed through crosslinking of RNase E
Source: EMBO J. 2016 Nov 11;36(3):374–87. doi: 10.15252/embj.201694639 (PMC5286369; doi:10.15252/embj.201694639)
Supplement: Supplementary file 7 — Table EV5 [file EMBJ-36-374-s008.docx]

Expanded View Table 5: Experimentally verified* sRNA-mRNA interactions recovered

| **sRNA** | **mRNA** | **mRNA seed start**** | **mRNA seed end**** | **ID** | **# unique hybrids** | **Score** |
| --- | --- | --- | --- | --- | --- | --- |
| MicA | *ompA* | 1149530 | 1149546 | 1663730-1_4 | 26 | 6.6 |
| MicM (ChiX) | *chbC* | 2420775 | 2420796 | 614692-2_6 | 36 | 6.6 |
| DsrA | *hns* | 1737949 | 1737962 | 1595555-1_4 | 14 | 6.4 |
| MicA | *phoP* | 1612165 | 1612188 | 1450114-1_4 | 4 | 5.4 |
| RyhB | *yadR (erpA)* | 180797 | 180823 | 1317348-1_4 | 12 | 5.2 |
| RprA | *csgD* | 1462420 | 1462435 | 3130985-1_2 | 9 | 3.9 |
| IsrA (McaS) | *csgD* | 1462417 | 1462433 | 2134250-1_3 | 6 | 3.6 |
| FnrS | *iscR* | 3382723 | 3382750 | 5554480-1_1 | 2 | 3.2 |
| RyhB | *shiA* | 2723041 | 2723092 | 418855-1_8 | 1 | 3.1 |
| RybB | *ompC* | 3045740 | 3045790 | 5462902-1_1 | 1 | 3.1 |
| DsrA | *rpoS* | 3588171 | 3588194 | 6390122-1_1 | 1 | 3.1 |
| OmrA | *csgD* | 1462397 | 1462416 | 1022537-1_5 | 2 | 1.2 |
| RyhB | *cysE* | 4529386 | 4529399 | 5826677-1_1 | 2 | 1.2 |
| MicM (ChiX) | *citA* | 733553 | 733565 | 5189839-1_1 | 1 | 1.1 |

*Interactions were collated from sRNATarBase 3.0 (Wang *et al* 2015). The coordinates were checked relative to the original literature and inaccurate seed coordinates corrected where necessary. Supplementary Table 4 listed the coordinates that were found to be inaccurate and the corrected coordinates. Seed sequences were converted to coordinates in the *E. coli* O157:H7 str. Sakai genome using BLAST and verified using IntaRNA. The total list of experimentally verified sRNA-mRNA interactions for both *E. coli* K12 and *E. coli* O157:H7 str. Sakai are presented in Table EV3.

**Coordinates for mRNA seed start and end are for *E. coli* O157:H7 str. Sakai (GeneBank Acc# NC_002695.1).

Wang J, Liu T, Zhao B, Lu Q, Wang Z, Cao Y, Li W. sRNATarBase 3.0: an updated database for sRNA-target interactions in bacteria. Nucleic Acids Res. 2015 Oct 25.
