# Supplementary material for: Small RNA interactome of pathogenic E. coli revealed through crosslinking of RNase E
Source: EMBO J. 2016 Nov 11;36(3):374–87. doi: 10.15252/embj.201694639 (PMC5286369; doi:10.15252/embj.201694639)
Supplement: Supplementary file 10 — Table EV8 [file EMBJ-36-374-s011.docx]

Expanded View Table 8: Strains, plasmids, and primers used in this study.

| **STRAINS** |  |  |  |  |
| --- | --- | --- | --- | --- |
| **Serotype** | **Strain** | **Genotype** | **Strain ID** | **Reference** |
| *E. coli* O157:H7 | Sakai | *∆stx1 stx2A::kan*, Kan^R^ | JJT163 | (Dahan *et al*, 2004) |
|  | *hfq-HTF* | *hfq-*HTF, His-TEV-FLAG tagged Hfq | JJT101 | (Tree *et al*, 2014) |
|  | *rne-HTF* | *rne-*HTF, His-TEV-FLAG tagged RNase E | JJT204 | This study |
|  | ZAP198 |  |  |  |
|  | *∆esr41* | ZAP198 ∆*esr41* |  | This study |
| *E. coli* K12 | DH5α | F^-^ endA1 glnV44 thi-1 recA1 relA1 gyrA96 deoR nupG Φ80d*lacZ*ΔM15 Δ(*lacZYA-argF*)U169, hsdR17(r_K_^-^ m_K_^+^), | JJT073 | (Taylor *et al*, 1993) |
|  | Top10F’ | F'[lacI^q^ Tn10(tet^R^)] mcrA Δ(mrr-hsdRMS-mcrBC) φ80lacZΔM15 ΔlacX74 deoR nupG recA1 araD139 Δ(ara-leu)7697 galU galK rpsL(Str^R^) endA1 | JJT064 | Invitrogen |
|  | N3433 (CSGC #6976) |  | JJT268 | (9) |
|  | N3431 (CSGC #6975) | *rne-3071* | JJT269 | (9) |
|  |  |  |  |  |
| **PLASMIDS** | **Plasmid** | **Description** | **Stock ID** | **Reference** |
|  | p3Z/Col1A | Colicin 1A expression construct |  | (Salvail *et al*, 2013; Brickman & Armstrong, 1996) |
|  | p3Z/ColB | Colicin B expression construct |  | (Salvail *et al*, 2013; Brickman & Armstrong, 1996) |
|  | pXG10SF | For translation fusions with superfolder GFP, Cm^R^ | JJT014 | (Corcoran *et al*, 2012) |
|  | pXG10SF::HdeA | *hdeA* -51🡪+117 fused to sfGFP, Cm^R^ | JJT256 | This study |
|  | pXG10SF::HdeA-M1a | Point mutant for RyhB-M1a, Cm^R^ | JJT264 | This study |
|  | pXG10SF::HdeA-M1b | Point mutant for GadY-M1, Cm^R^ | JJT265 | This study |
|  | pXG10SF::RssA (YchK) | RssA -100🡪+150 fused to sfGFP, Cm^R^ | JJT136 | This study |
|  | pXG10SF::RssA-M1 | Point mutant for RyeB-M1, Cm^R^ | JJT157 | This study |
|  | pXG10SF::ZapB(YiiU) | ZapB -136🡪+153 fused to sfGFP, Cm^R^ | JJT135 | This study |
|  | pXG10SF::ZapB-M1 | Point mutant for RyhB-M1b, Cm^R^ | JJT158 | This study |
|  | pXG10SF::FrdA | *frdA* -94 🡪 +90 fused to sfGFP, Cm^R^ | JJT137 | This study |
|  | pXG10SF::FrdA-M1 | Point mutant for RyhB-M1c, Cm^R^ | JJT159 | This study |
|  | pXG10SF::CirA | *cirA* -160🡪+60 fused to sfGFP, Cm^R^ | JJT233 | This study |
|  | pXG10SF::CirA-M1 | Point mutant for Esr41-M1, Cm^R^ | JJT287 | This study |
|  | pXG10SF::ChuA | *chuA* +361🡪 +1980 fused to sfGFP, Cm^R^ | JJT091 | This study |
|  | pXG10SF::ChuA-M1 | Point mutant for Esr41-M1, Cm^R^ | JJT160 | This study |
|  | pXG10SF::Bfr | *bfr* -24 🡪 +48 fused to sfGFP, Cm^R^ | JJT231 | This study |
|  | pXG10SF::Bfr-M1 | Point mutant for Esr41-M2, Cm^R^ | JJT161 | This study |
|  | pJV300 | Scrambled sRNA control vector, Amp^R^ | JJT034 | (Urban & Vogel, 2007) |
|  | pZE12::*luc* | Constitutive sRNA expression vector, Amp^R^ | JJT032 | (Urban & Vogel, 2007) |
|  | pZE12::*ryhB* | RyhB expression construct, Amp^R^ | JJT224 | This study |
|  | pZE12::*ryhB-M1a* | Point mutant for HdeA-M1a, Amp^R^ | JJT162 | This study |
|  | pZE12::*ryhB-M1b* | Point mutant for ZapB-M1, Amp^R^ | JJT158 | This study |
|  | pZE12::*ryhB-M1c* | Point mutant for FrdA-M1, Amp^R^ | JJT159 | This study |
|  | pZE12::*ryeB* | RyeB expression construct, Amp^R^ | JJT223 | This study |
|  | pZE12::*ryeB-M1* | Point mutant for RssA-M1, Amp^R^ | JJT246 | This study |
|  | pZE12::*gadY* | GadY expression construct, Amp^R^ | JJT111 | This study |
|  | pZE12::*gadY-M1* | Point mutant for HdeA-M1b, Amp^R^ | JJT245 | This study |
|  | pZE12::*esr41* | Esr41 expression construct, Amp^R^ | JJT228 | This study |
|  | pZE12::*esr41-M1* | Point mutant for ChuA-M1 and CirA-M1, Amp^R^ | JJT249 | This study |
|  | pZE12::*esr41-M2* | Point mutant for Bfr-M1, Amp^R^ | JJT250 | This study |
|  | pTOF25 | Allelic exchange vector | JJT024 | (Merlin *et al*, 2002) |
|  | pTOF25::*rne*-HTF<>tetRNA | Allelic exchange vector for inserting HTF tag into chromosomal *rne* | JJT277 | This study |
|  |  |  |  |  |
| **PRIMERS** | **Primer** | **Sequence** | **Purpose** | |
| For constructs | hdeA.NsiI.F | tttttATGCATgtcgaaattgattcgtgacg | HdeA fusion | |
|  | hdeA.NheI.R | tttttGCTAGCACAGGTCCAGGAGTTGACCGG | HdeA fusion | |
|  | ychK.NsiI.F | tttttATGCATactaccacagggacaaagctg | YchK fusion | |
|  | ychK.NheI.R | tttttGCTAGCAACGATATCAATTTCAATACC | YchK fusion | |
|  | yiiU.NsiI.F | tttttATGCATgccgctggacagcgatggcg | YiiU fusion | |
|  | yiiU.NheI.R | tttttGCTAGCCAGCTCTTCGCGCTGATGCTG | YiiU fusion | |
|  | frdA.NsiI.F | tttttATGCATaaaggagcagtggaatagcg | FrdA fusion | |
|  | frdA.NheI.R | tttttGCTAGCATTTGGATTTGCCTGCGCGGC | FrdA fusion | |
|  | cirA.NsiI.F | tttttATGCATAatcaaaaaaggctgacaaatc | CirA fusion | |
|  | cirA.NheI.R | tttttGCTAGCAATAGCGGACAAACACAGCCCGAC | CirA fusion | |
|  | bfr.NsiI.F | tttttATGCATtagtggaagcggagggactata | Bfr fusion | |
|  | bfr.NheI.R | tttttGCTAGCTCCCAATAGTTTATTGAGATA | Bfr fusion | |
|  | Esr41.ZE12.F | GATGCTCTAGGCATCACATTTTCTCCATGGGGTATTCCCTCCGCCGGCACTATGTGTTGCTGGCGTTTTTTTAT GGCATCAAATAAAACGAAAG | Esr41 expression construct | |
|  | ZE12.5P.R | 5P-GTGCTCAGTATCTTGTTATCC | Esr41 expression construct | |
|  | gadY.F | ACTGAGAGCACAAAGTTTCCCG | sRNA cloning | |
|  | gadY.XbaI.R | aaaaaTCTAGAAAAAAAACCCGGCATAGGGGACCGGGAA | sRNA cloning | |
|  | ryeB.F | GGCAAGGCAACTAAGCCTGCATTAATGC | sRNA cloning | |
|  | ryeB.XbaI.R | aaaaaTCTAGAAAAAAGAGACCGAACACGATTCCTG | sRNA cloning | |
|  | ryhB.F | GCGATCAGGAAGACCCTCGC | sRNA cloning | |
|  | ryhB.XbaI.R | aaaaaTCTAGAAAAAGCCAGCACCCGGCTGG | sRNA cloning | |
|  | ZE-CAT | TGGGATATATCAACGGTGGT | sRNA cloning | |
|  | JVO-0155 | CCGTATGTAGCATCACCTTC | sRNA cloning | |
|  | PLlacOC | 5P-GTGCTCAGTATCTTGTTATC | sRNA cloning | |
|  | PLlacOB | CGCACTGACCGAATTCATTAA | sRNA cloning | |
|  | ZE12.seq.F | CTCGAGAATTGTGAGCGGATAAC | For sequencing ZE12 constructs | |
|  | ZE12.seq.R | CAGTCTTTCGACTGAGCCTTTCG | For sequencing ZE12 constructs | |
|  | rne.5.NotI.F | gaaaaaGCGGCCGCCGATTGTACGTCCACAAGATG | Construction of rne-HTF allelic exchange vector | |
|  | rne_5.BamHI_link.R | CCGTTCCAAGGATCCAGcAGagcgCTCAACAGGTTGCGGACGCGCAG | Construction of rne-HTF allelic exchange vector | |
|  | rne.3.BamHI_link.F | cgctctgctggatccttggaacggTAATTAGCTCAAGTAATCAAGC | Construction of rne-HTF allelic exchange vector | |
|  | rne.3.NotI.R | gaaaaaGCGGCCGCCAGGTCGATGCTTCGCGTTCG | Construction of rne-HTF allelic exchange vector | |
|  | rne.ext.F | TGGCCTCTGGCAAAGTCTGG | Screening rne-HTF insertion | |
|  | rne.ext.R | TTGCTGAATCTGGCGAACACC | Screening rne-HTF insertion | |
|  |  |  |  | |
| For site directed mutagenesis | hdeA.M1.ryhB.F | GCCAGTTGT**T**AG**T**AATGCAG | HdeA-M1a mutant | |
|  | hdeA.M1.ryhB.R | CTGCATTACTAACAACTGGC | HdeA-M1a mutant | |
|  | hdeA.M1.gadY.F | TTATTCTTGG**C**GG**C**CTGCTTCTTC | HdeA-M1b mutant | |
|  | hdeA.M1.gadY.R | GAAGAAGCAGGCCGCCAAGAATAA | HdeA-M1b mutant | |
|  | ryhB.M1.hdeA.F | GCACGACATT**A**CT**A**ACATTGCTTC | RyhB-M1a mutant | |
|  | ryhB.M1.hdeA.R | GAAGCAATGTTAGTAATGTCGTGC | RyhB-M1a mutant | |
|  | gadY.M1.hdeA.F | GAGACGGCAG**G**CT**G**TCCTCTTCCC | GadY-M1 mutant | |
|  | gadY.M1.hdeA.R | GGGAAGAGGACAGCCTGCCGTCTC | GadY-M1 mutant | |
|  | ychK.M1.RyeB.F | TAGGGCTGGC**C**CT**C**GGATCTGGCG | YchK-M1 mutant | |
|  | ychK.M1.RyeB.R | CGCCAGATCCGAGGGCCAGCCCTA | YchK-M1 mutant | |
|  | ryeB.M1.ychK | TGCCAACTTT**G**AG**G**GCACGGCTCT | RyeB-M1 mutant | |
|  | ryeB.M1.ychK.R | AGAGCCGTGCCCTCAAAGTTGGCA | RyeB-M1 mutant | |
|  | ryhB.M1.yiiU.F | CATTGCTCACAT**G**GCTTC**G**AGTATTACTTAG | RyhB-M1b mutant | |
|  | ryhB.M1.yiiU.R | CTAAGTAATACTCGAAGCCATGTGAGCAATG | RyhB-M1b mutant | |
|  | yiiU.M1.ryhB.F | TTGAGAAACT**C**GAAGC**C**AAAGTACAGC | YiiU-M1 mutant | |
|  | yiiU.M1.ryhB.R | GCTGTACTTTGGCTTCGAGTTTCTCAA | YiiU-M1 mutant | |
|  | frdA.M1.ryhB.F | ctggaggaatcagGTGCAAACCT | FrdA-M1 mutant | |
|  | frdA.M1.ryhB.R | AGGTTTGCACctgattcctccag | FrdA-M1 mutant | |
|  | ryhB.M1.frdA.F | CTGAAAGCAC**CTG**ATTGCTCACATTG | RyhB-M1c mutant | |
|  | ryhB.M1.frdA.R | CAATGTGAGCAATCAGGTGCTTTCAG | RyhB-M1c mutant | |
|  | esr41.M1.cirA.F | CATTTTCTCCATCTCGTATTCCCTC | Esr41-M1 mutant | |
|  | esr41.M1.cirA.R | GAGGGAATACGAGATGGAGAAAATG | Esr41-M1 mutant | |
|  | chuA.M1.F | gcattttttattttcGAGatggagaatcg | ChuA-M1 mutant | |
|  | chuA.M1.R | cgattctccatCTCgaaaataaaaaatgc | ChuA-M1 mutant | |
|  | bfr.M1.F | gtggaagcggGAggactataaa | Bfr-M1 mutant | |
|  | bfr.M1.R | tttatagtccTCccgcttccac | Bfr-M1 mutant | |
|  | esr41.M1.bfr.F | GGGTATTCCTCCCGCCGGCAC | Esr41-M2 mutant | |
|  | esr41.M1.R | GTGCCGGCGGGAGGAATACCC | Esr41-M2 mutant | |
|  |  |  |  | |
| CLASH library preparation | P5 | AATGATACGGCGACCACCGAGATCTACACTCTTTCCCTACACGACGCTCTTCCGATCT | Forward primer to amplify RACE products and CRAC cDNA libraries | |
|  | PE_miRCat | CAAGCAGAAGACGGCATACGAGATCGGTCTCGGCATTCCTGGCCTTGGCACCCGAGAATTCC | To amplify CRAC cDNA libraries | |
|  | L5 linker | 5’invddT-ACACrGrArCrGrCrUrCrUrUrCrCrGrArUrCrU-barcode | 5’ linker for RLM-RACE and CRAC. barcode = 2-11nt of unique sequence | |
| QPCR primers | sfGFP.F | GTTCCATGGCCAACACTTGTCACT | sfGFP QPCR | |
|  | sfGFP.R | TACATAACCTTCGGGCATGGCACT | sfGFP QPCR | |
|  | 16S.F | ATTGACGTTACCCGCAGAAG | 16S QPCR | |
|  | 16S.R | CGCTTTACGCCCAGTAATTC | 16S QPCR | |
| Northern probe | 5S rRNA | GTTTCACTTCTGAGTTCGGCATGGGGTCAG | 5S rRNA probe | |
|  |  |  |  | |
| **SEQUENCE** | His-TEV-FLAG tag | GGATCCATGGAGCACCATCACCATCACCATGATTATGATATTCCAACTACTGCTAGCGAGAATTTGTATTTTCAGGGTGAGCTCGACTACAAAGATGACGACGATAAAGACTACAAAGATGACGACGATAAAGACTACAAAGATGACGACGATAAATGAGAATTC | GenBank Acc# KX714724 | |

1. Dahan S, Knutton S, Shaw RK, Crepin VF, Dougan G & Frankel G (2004) Transcriptome of enterohemorrhagic *Escherichia coli* O157 adhering to eukaryotic plasma membranes. *Infect Imm* 72: 5452–5459

2. Tree JJ, Granneman S, McAteer SP, Tollervey D, Gally DL (2014) Identification of Bacteriophage-Encoded Anti-sRNAs in Pathogenic Escherichia coli. *Mol Cell* 55(2):199–213.

3. Taylor RG, Walker DC, McInnes RR (1993) E. coli host strains significantly affect the quality of small scale plasmid DNA preparations used for sequencing. *Nucleic Acids Res* 21(7):1677–1678.

4. Salvail H, Caron M-P, Bélanger J, Massé E (2013) Antagonistic functions between the RNA chaperone Hfq and an sRNA regulate sensitivity to the antibiotic colicin. *EMBO J* 32(20):2764–78.

5. Brickman TJ, Armstrong SK (1996) Colicins B and Ia as novel counterselective agents in interspecies conjugal DNA transfers from colicin-sensitive escherichia coli donors to other gram-negative recipient species. *Gene* 178(1-2):39–42.

6. Corcoran CP, et al. (2012) Superfolder GFP reporters validate diverse new mRNA targets of the classic porin regulator, MicF RNA. *Mol Microbiol* 84(3):428–445.

7. Urban JH, Vogel J (2007) Translational control and target recognition by Escherichia coli small RNAs in vivo. *Nucleic Acids Res* 35(3):1018–1037.

8. Merlin C, McAteer S, Masters M (2002) Tools for characterization of Escherichia coli genes of unknown function. *J Bacteriol* 184(16):4573–4581.
