# Supplementary figures and images for: Small RNA interactome of pathogenic E. coli revealed through crosslinking of RNase E
Source: EMBO J. 2016 Nov 11;36(3):374–87. doi: 10.15252/embj.201694639 (PMC5286369; doi:10.15252/embj.201694639)

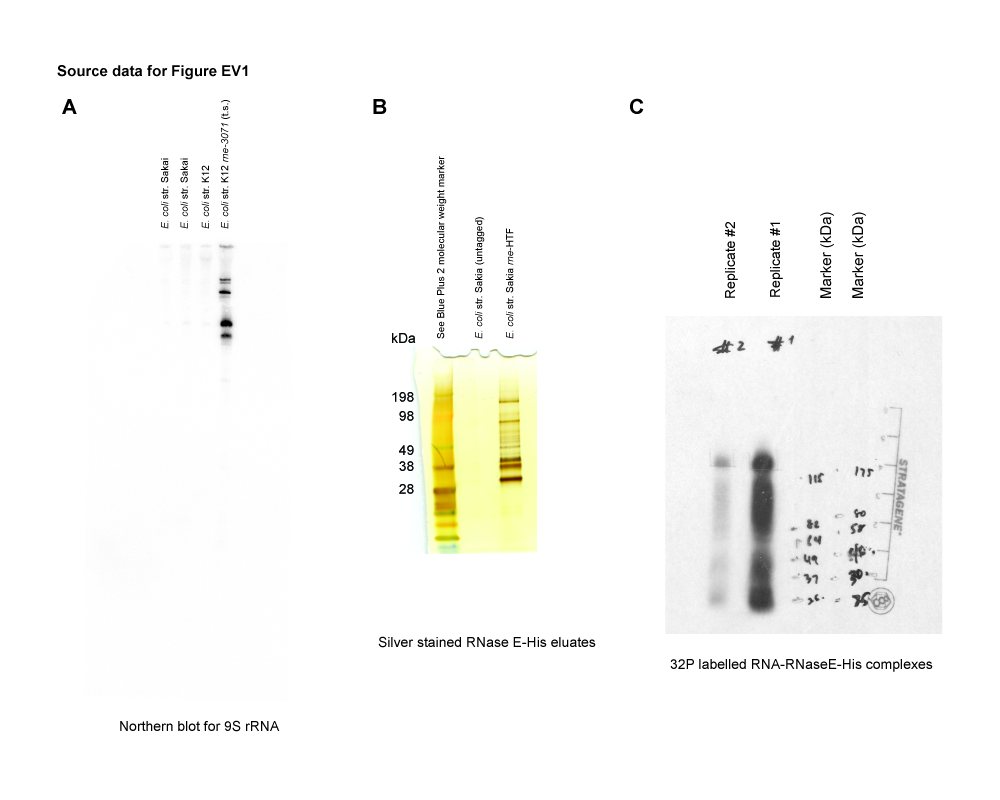

Supplement: Supplementary file 11 — Source Data for Figure EV1 [file EMBJ-36-374-s003.zip › embj201694639-sup-0003-SDataEV.tif]
